# Atypic Retinitis Pigmentosa Clinical Features Associated with a Peculiar CRX Gene Mutation in Italian Patients

**DOI:** 10.3390/medicina60050797

**Published:** 2024-05-11

**Authors:** Marco Piergentili, Vito Spagnuolo, Vittoria Murro, Dario Pasquale Mucciolo, Dario Giorgio, Ilaria Passerini, Elisabetta Pelo, Fabrizio Giansanti, Gianni Virgili, Andrea Sodi

**Affiliations:** 1 Eye Clinic, Neuromuscular and Sense Organs Department, Careggi University Hospital, 50134 Florence, Italy; vitospa93@gmail.com (V.S.); vittoria.murro@unifi.it (V.M.); dario.mucciolo@gmail.com (D.P.M.); dario.giorgio87@gmail.com (D.G.); fabrizio.giansanti@unifi.it (F.G.); gianni.virgili@unifi.it (G.V.); 2 Department of Neurosciences, Psychology, Drug Research and Child Health (NEUROFARBA), University of Florence, 50139 Firenze, Italy; 3 Department of Ophthalmology, Pistoia Hospital, 51100 Pistoia, Italy; 4 Department of Ophthalmology, Livorno Hospital, 57124 Livorno, Italy; 5 Department of Genetic Diagnosis, Careggi University Hospital, 50134 Firenze, Italy; ilariapasserini70@gmail.com (I.P.); peloe@aou-careggi.toscana.it (E.P.)

**Keywords:** *CRX* gene, late-onset retinal dystrophy, pericentral retinitis pigmentosa, sector retinitis pigmentosa, genotype

## Abstract

*Purpose*: To describe an atypical phenotypic pattern of late-onset retinitis pigmentosa (RP) due to the same specific c.425A>G (p.Tyr142Cys) heterozygous mutation in the cone–rod homeobox gene (*CRX* gene) in two unrelated Italian patients. *Case 1*: A 67-year-old woman (P.P.) was incidentally diagnosed with sector RP at the age of 50. The patient was initially asymptomatic and did not have any family history of retinal dystrophy. Fundus examination showed the presence of typical retinal pigmentary deposits with a peculiar pericentral/sector distribution. Genomic sequencing disclosed the missense mutation c.425A>G (p.Tyr142Cys) in the *CRX* gene. During the follow-up period of 7 years, the patient maintained good visual acuity and complained only of mild symptoms. *Case 2*: A 76-year-old man (P.E.) presented with nyctalopia and visual field constriction since the age of 50. Fundus examination showed the presence of retinal pigment deposits with a concentric pericentral and perimacular pattern. A full-field electroretinogram (ffERG) showed extinguished scotopic responses and reduced abnormal photopic and flicker cone responses. Genomic sequencing identified the same missense mutation, c.425A>G (p.Tyr142Cys), in the *CRX* gene. Similarly to the first case, during the whole follow-up of 7 years, the visual acuity remained stable, as did the visual field and the patient’s symptoms. *Conclusions*: We report the first cases of late-onset retinitis pigmentosa related to a specific heterozygous *CRX* gene mutation in exon 4. We also report two atypical phenotypic RP patterns related to mutations in the *CRX* gene.

## 1. Introduction

Retinitis pigmentosa (RP, MIM 268000), with an estimated prevalence of about 1/3000–1/5000, represents the most common hereditary retinal dystrophy. It refers to a group of rod–cone dystrophies and is characterized by the loss of retinal photoreceptors, leading to progressive visual decline [1]. RP may follow various patterns of inheritance: autosomal dominant (AD), autosomal recessive (AR), or X-linked. Rarely, the genetic pattern follows a mitochondrial mode of transmission. Additionally, about 40–50% of cases present de novo mutations in the absence of a positive family history of RP, and such cases are known as sporadic RP [2].

Non-syndromic RP is genetically very heterogeneous, and so far, mutations in over 80 genes or loci have been identified as pathogenic [3].

The *CRX* gene (cone–rod homeobox gene; OMIM 120970) is located on chromosome 19q13.33, and it encodes for a protein containing 299 amino acids, which represents a transcription factor involved in controlling the expression of other photoreceptor-specific genes. It is predominantly expressed in photoreceptors, where it plays a crucial role in their differentiation and integrity through interaction with other transcription factors (NRL, RAX, and NR2E3) [4,5].

Because *CRX* appears to interact with many retinal genes, mutations in the *CRX* gene are responsible for a wide phenotypic spectrum of retinal dystrophies, including cone–rod dystrophy type 2 (CORD2), Leber congenital amaurosis (LCA), pattern dystrophy (PD), macular degeneration, and RP [4,6].

To date, around 49 mutations of the *CRX* gene, comprising 86 alleles, have been documented, contributing to 4.76% of cone–rod dystrophy cases, 2.35% of LCA cases, and 0.80% of RP cases. Most mutations arise de novo, and the dysfunctional mutant CRX protein interferes with the function of the wild-type CRX protein, acting with a dominant negative effect [7,8,9].

In most cases, the inheritance pattern for CRX-associated retinopathies is autosomal dominant, even if incomplete penetrance and variable expressivity have been previously reported [10].

The typical clinical signs of RP include retinal pigmentation in the form of bone spicules, vascular attenuation, and waxy pallor of the optic disc. Typical symptoms at presentation are nyctalopia and visual field constriction. The disease is often diagnosed in children or young adults, but more rarely, it may have a late onset (sometimes around the fifth decade or later). The rate of disease progression often depends on the age of presentation of the disease, which is usually faster in cases of early onset [11].

In this case series, we describe two cases of late-onset atypical RP associated with the c.425A>G (p.Tyr142Cys) mutation in the heterozygosity of the *CRX* gene at the level of exon 4. This mutation has been previously reported in the literature, causing a flecked-retina phenotypic pattern of Leber congenital amaurosis/early-onset retinal dystrophy [12]. The broad clinical and genetic heterogeneity associated with the *CRX* gene poses a diagnostic challenge both for the clinician and geneticist, thus necessitating an analysis based on genotype–phenotype correlation.

## 2. Methods

Following informed consent and pre-test genetic counseling, 10 mL of peripheral blood were obtained from the antecubital vein using EDTA-containing vials. DNA was extracted from 200 μL of peripheral blood by using automated DNA extractors: BioRobot EZ1 (QIAGEN, Hilden, Germany) or QIAsymphony SP workstation (QIAGEN GmbH, Germany), according to the manufacturer protocols.

Genomic DNA from patients and available family members was used for identifying mutations by a targeted NGS gene panel for IRDs covering 104 genes. Panel-based testing for IRD genes was conducted for these patients on the Illumina NextSeq TM500 platform (Illumina, San Diego, CA, USA). Variations were annotated using Alissa Interpret Rev. 5.4.2 Agilent Inc. (SeqOne, Montpellier, France) by comparing with other databases (1000 Genome Project, Exome Aggregation Consortium, Ensmble, dbSNP, ClinVar, Human Gene Mutation Database (HGMD), Variation Database (LOVD), RetinoGenetics, RetNet, Mutation Database of Retina International) and predicted for pathogenicity with online bioinformatic tools (SIFT, PolyPhen, Mutation Taster, Mutation Assessor, and Variant Effect Predictor). We evaluated allele frequencies (GnomAD), co-segregation analysis, and published data. The CRX variant is classified according to the current revised guidelines of ACMG and confirmed with Sanger sequencing.

## 3. Case 1

A 67-year-old woman (P.P.) was referred to our tertiary referral ocular genetics center (Careggi University Hospital, Florence, Italy) due to sector RP. The patient was initially asymptomatic, and the diagnosis was incidentally made at the age of 50. The patient was the only affected member of the family, and there was no family history of genetic eye conditions, and she denied consanguinity to her parents. Progeny did not show any signs or symptoms suggesting ocular diseases. When asked, she referred to some difficulty with night vision (nyctalopia). On examination, her best corrected visual acuity (BCVA) measured 0.0 with Thompson logMAR in both eyes and remained stable over a 7-year follow-up period. Color vision evaluated with Ishihara plates was also normal, as was her extraocular motility. During the follow-up period, no complications, such as typical lens opacities or macular edema, have occurred.

**Figure 1:**
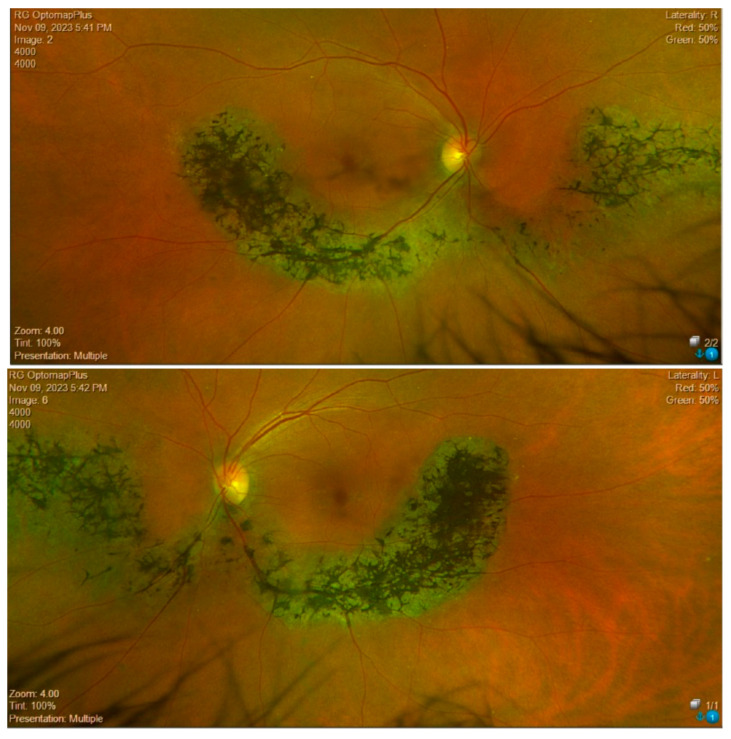
Wide field color fundus retinography (Optomap, Daytona, Nikon) of the right and left eyes of Case 1.

Fundus examination showed vascular attenuation and the presence of typical retinal pigmentary deposits in the form of bone spicules along the nasal and temporal inferior vascular arcades in both eyes (Figure 1). These findings are compatible with a sector RP, even if with a peculiar pericentral distribution of the retina alterations. Additionally, a chorioretinal flat nevus was noted superotemporally in the left eye.

Bilaterally, the macula showed an alteration of the foveal reflex with retinal pigment epithelium (RPE) dystrophy. Spectral-domain optical coherence tomography (SD-OCT, Spectralis Heidelberg Engineering, Heidelberg, Germany) centered at the fovea demonstrated inferior pericentral atrophy of the outer retinal layers with the absence of the IS-OS junction (ellipsoid zone) and RPE atrophy with foveal sparing of a diameter of 4736 microns in the right eye and 5133 microns in the left eye.

**Figure 2:**
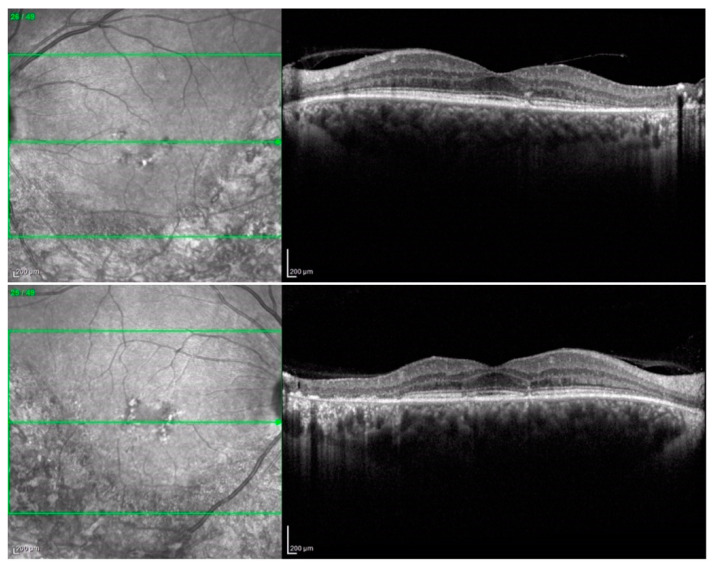
SD-OCT (Heidelberg Spectralis).

**Figure 3:**
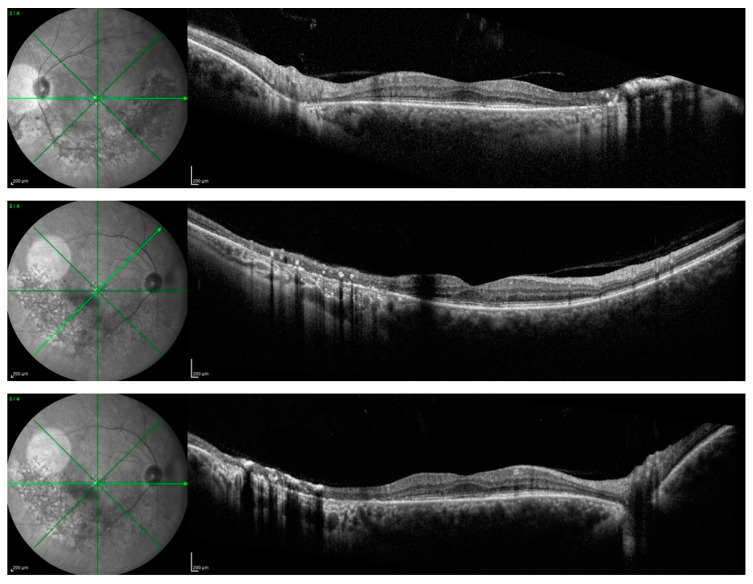
Wide-field SD-OCT shows pericentral atrophy of the outer retinal layers with the absence of the IS-OS junction (ellipsoid zone) and RPE atrophy with foveal sparing.

**Figure 4:**
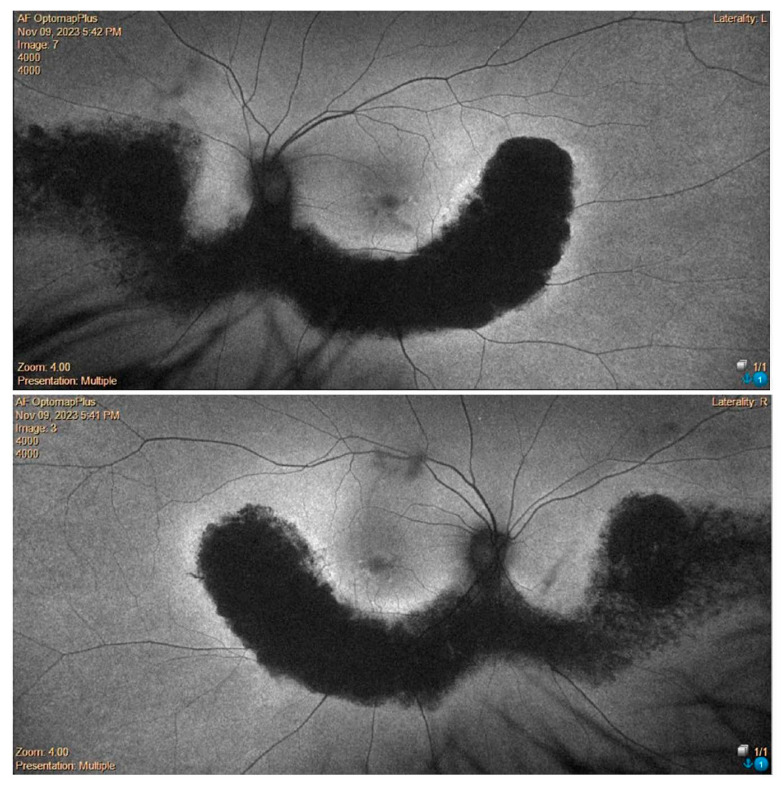
Wide-field green autofluorescence (GAF, Optomap) of the right and left eye.

**Figure 5:**
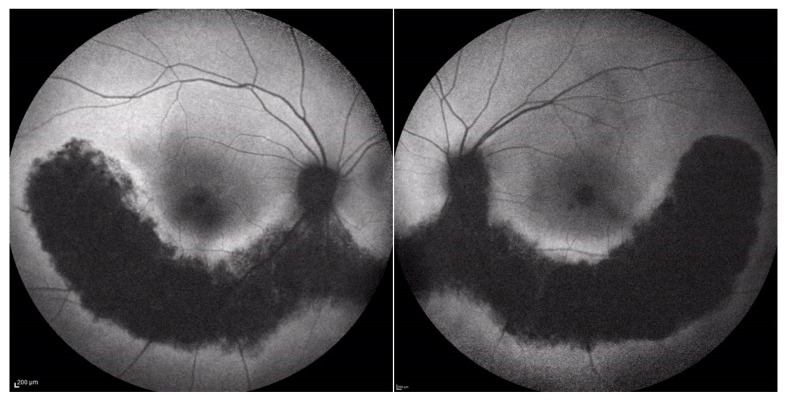
Blue autofluorescence (BAF, Heidelberg Spectralis).

Additionally, the OCT revealed the presence of a vitreomacular adhesion (VMA) in both eyes with a central foveal thickness (CFT) at the last follow-up of 211 microns in the right eye and 238 microns in the left eye (Figure 2 and Figure 3). Goldman visual field (GVF) demonstrated the presence of a superior arcuate scotoma corresponding to the abnormal retina without any progression throughout the entire follow-up period. Blue autofluorescence (BAF, Heidelberg Spectralis, Heidelberg Engineering) and green autofluorescence (GAF, Optomap, Daytona, Nikon) showed areas of hypo-autofluorescence along the inferior vascular arcades corresponding to the diseased and atrophic retina (Figure 4 and Figure 5).

Molecular genetic analysis for a panel of genes causing RP disclosed a heterozygous pathogenic c.425A>G (p.Tyr142Cys) *CRX* mutation in exon 4.

## 4. Case 2

A 76-year-old man (P.E.) was referred to our tertiary referral ocular genetics center (Careggi University Hospital, Florence, Italy) due to a suspected diagnosis of RP made at the age of 68. The onset of the symptoms started at the age of 49 and included nyctalopia and visual field constriction.

**Figure 6:**
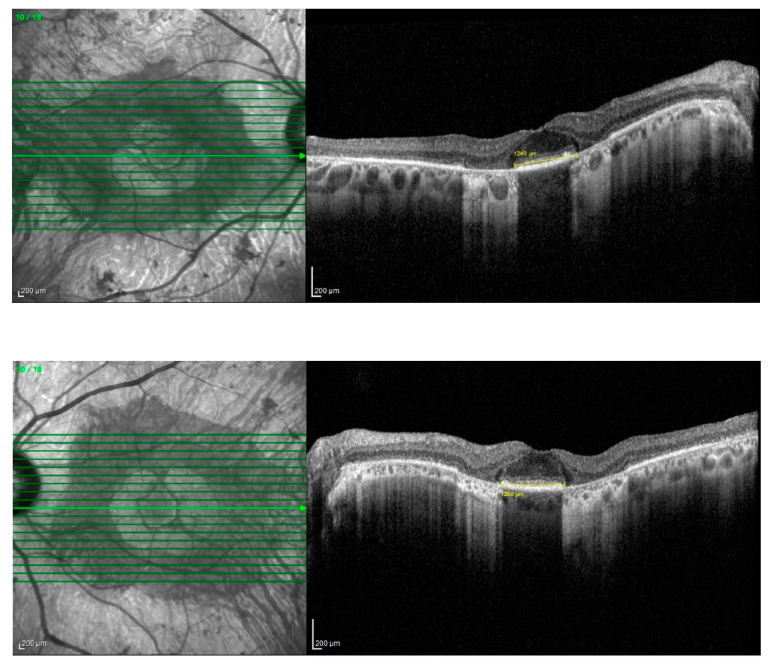
SD-OCT (Heidelberg, Spectralis).

**Figure 7:**
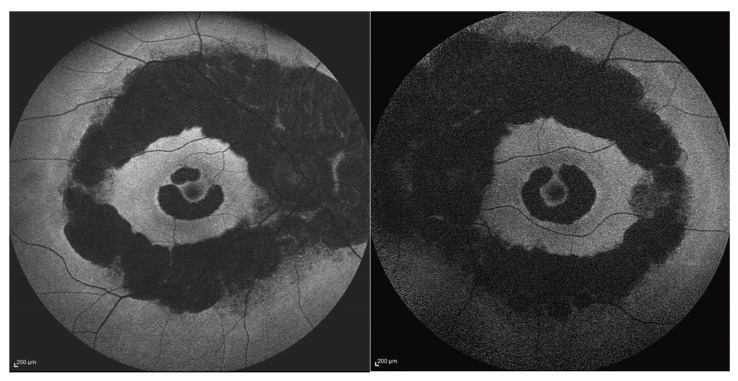
Fundus autofluorescence (FAF).

**Figure 8:**
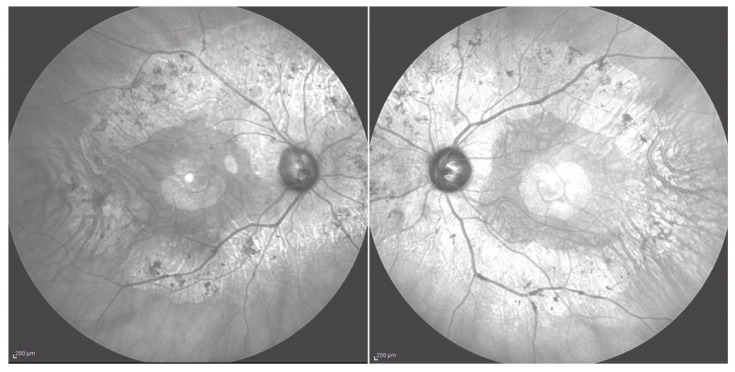
Infrared imaging (Heidelberg Spectralis).

There were no cases of other affected members in the pedigree, except for a deceased brother reported to have visual difficulties. However, no clinical data were available to raise the suspicion of any retinal dystrophy or eye disease. Efforts were made to obtain any clinical records on our systems or previous notes, which were unsuccessful. However, the patients (P.E.) did not report remembering the brother having problems navigating at night light. Additionally, the patient denied consanguinity between his parents, and the progeny did not show any ocular abnormalities, although none of them underwent molecular genetic testing. On examination, BCVA was 0.0 logMAR in both eyes on the first consultation and remained stable over the 7-year follow-up period. Extraocular motility and color vision with the Ishihara plates were also normal. Fundus examination with a slit lamp showed the presence of typical retinal pigment deposits in the form of bone spicules along the inferior and superior vascular arcades with a pericentral concentric pattern. Additionally, there was involvement of the peri-macular area with foveal sparing. In fact, an OCT examination of the macula showed peri-foveal atrophy of the outer retinal layers with the absence of the IS-OS junction and RPE atrophy. A small foveal area with a diameter of 1240 microns in the right eye and 1250 microns in the left eye was spared. This was in agreement with the excellent visual performance of our patient. Central foveal thickness (CFT) at the last OCT examination was 258 microns in the right eye and 259 microns in the left one (Figure 6). Fundus autofluorescence (BAF and GAF) showed areas of hypo-autofluorescence compatible with the diseased and atrophic retina identified on OCT in a specific ‘double ring’ pattern appearance (Figure 7). The infrared module also showed the same characteristic pattern due to the involvement of the deep retinal structures at the level of the RPE (Figure 8).

Electrodiagnostic testing (ffERG) showed extinguished scotopic responses and reduced abnormal photopic and flicker cone responses. GVF repeated during the follow-up period showed a concentric ring scotoma within the central 30° of the visual field.

Also, in this case, molecular genetic analysis for a panel of genes causing RP disclosed a pathogenic heterozygous c.425A>G (p.Tyr142Cys) *CRX* mutation in exon 4.

## 5. Discussion

Retinitis pigmentosa (RP) refers to a group of inherited disorders in which abnormalities of the photoreceptors (rods and cones) of the retina lead to progressive visual loss. Most of the genes associated with RP encode proteins involved in phototransduction, the visual cycle (production and recycling of the rhodopsin chromophore), photoreceptor structure, or photoreceptor gene transcription [13,14]. The *CRX* gene (19q13.3) encodes a homeobox transcription factor critical for the differentiation of cones and rods and the maintenance of their normal function [15,16]. The protein structure has three domains: the homeodomain (which binds the DNA of other retinal genes), the WSP domain, and the OTX domain. Missense mutations are confined within the homeodomain, while frame-shift mutations leading to premature stop codons are limited to the OTX domain. However, both types of mutations have a dominant negative effect on retinal development, hindering the biogenesis of photoreceptor outer segments and thus resulting in early photoreceptor degeneration [9].

Mutations in the *CRX* gene may be associated with various phenotypic presentations within the spectrum of inherited retinal dystrophies, and sometimes this may be probably related to the interaction of *CRX* with many other retinal genes.

*CRX* genetic variants may be responsible for different clinical phenotypes, including cone–rod dystrophy, Leber congenital amaurosis (LCA), macular degeneration, and RP [5].

One study reported that all mutations within the homeodomain were missense mutations, with most showing a phenotype of cone–rod dystrophy or macular degeneration. On the other hand, most mutations in other regions of the gene were frame-shift mutations resulting in truncating proteins (88%), associated with different phenotypic presentations (cone–rod dystrophy and macular degeneration in 36%, Leber congenital amaurosis in 40%, and RP in 24%) [17]. Moreover, most mutations occur de novo in the absence of a positive family history [8].

Both patients in our study received a diagnosis of RP around the fifth decade of life, with mild symptoms and excellent central visual acuity. This presentation is compatible with a late-onset RP, where the attenuated signs and symptoms of the disease may be detected after middle age. It has been described in the literature that autosomal dominant mutations in the CRX gene are responsible for late-onset RP, with reduced visual field and ERG amplitude and typical retinal pigment deposits presenting during the fifth and sixth decades of life [18]. However, contrary to previous reports, our cases did not show a significant clinical progression of the disease during the 7-year follow-up period. Furthermore, both patients exhibited a peculiar subtype associated with limited involvement of the fundus and foveal sparing. Pericentral RP has been characterized as a subtype of RP where, rather than the pathology initiating in the mid-periphery as in typical RP, the disease starts in the near periphery close to the vascular arcades and tends to preserve the far periphery [19].

In our study, the first patient (P.P.) showed a retinal alteration along the nasal and temporal inferior vascular arcades in both eyes. The second patient (P.E.) presented a more widespread pericentral involvement of both retinas along the superior and inferior vascular arcades associated with perimacular degeneration (Figure 4 and Figure 7).

Full-field electroretinogram (ffERG) in pericentral RP and sector RP is usually only mildly affected, showing a slight reduction in the scotopic and normal photopic responses [20,21,22]. The GVF, in this case, also showed a more extensive involvement with a ring scotoma within the central 30° of the visual field. OCT macula analysis showed the absence of the IS-OS junction in the involved retinal areas, with varying degrees of foveal sparing in our cases.

Genetic analysis of both our patients revealed the presence of a heterozygous mutation c.425A>G (p.Tyr142Cys) in *CRX* at exon 4. This mutation has been previously reported in the literature, causing a flecked-retina phenotypic pattern of Leber congenital amaurosis/early-onset retinal dystrophy [12]. This variable spectrum of clinical manifestations demonstrates the wide phenotypic variability associated with *CRX* gene mutations and the complex genotype–environment interaction. Because the CRX gene encodes for a transcription factor that binds and transactivates several photoreceptor-specific genes, this could be the reason for the different phenotypes associated with the same pathogenic mutation. Also, CRX acts synergistically with other transcription factors, such as NRL, RORB, and RAX, controlling the expression of other photoreceptor-specific genes. Additionally, the probable existence of genetic modifiers with a pleiotropic effect could have resulted in variable clinical outcomes, as previously described in HK1-related retinal dystrophies [23]. Ultimately, we cannot exclude the presence of undetected genetic variants in other genes as being responsible for the phenotypic variability.

In both our families, the reported patients were the only confirmed affected members. All other family members, to date, are unaffected. Presumably, in both our cases, a de novo mutation of the CRX gene occurred and caused a late-onset sporadic RP [24]. However, we cannot exclude the deceased brother of patient 2 from being affected by a mild form of retinal dystrophy, and this is a limitation of this study. As far as we know, we reported the first two cases of late-onset RP related to this specific *CRX* gene mutation. We also report two atypical RP phenotypic patterns of pericentral and sector RP related to mutations in the *CRX* gene. Previously, Matsui et al. described a case of pericentral involvement in a patient with CRX-related RP. However, the case described by Matsui and colleagues had different characteristics from ours: age (34 years old), reduced visual acuity, and different genetics. Also, differently, our first patient presented with a peculiar sectoral retinal involvement with a pericentral pattern. The absence of signs and symptoms in the offspring could indicate an incomplete penetrance of inheritance, which has been previously suggested in autosomal dominant forms of cone–rod dystrophies associated with *CRX* gene mutations [10].

A careful follow-up of the offspring could reveal the possibility of a late-onset autosomal dominant RP phenotype or incomplete penetrance, but we cannot exclude, in some cases, the development of more severe clinical pictures of retinal degeneration. In fact, the association of *CRX* mutations, which may cause severe forms of retinal degeneration like LCA, with apparently mild clinical pictures like sector or pericentral RP may be of interest for correct genetic counseling. Furthermore, in the future, the understanding of gene interactions leading to such different clinical phenotypes could allow the development of treatments targeting the specific underlying mechanism. In fact, the presence of additional genetic modifiers could lead to the failure of a possible replacement for gene therapy. The early detection of such different clinical scenarios, ranging from LCA to late-onset RP, would also be crucial to defining a prognosis and selecting patients after the development of new treatments.

## 6. Conclusions

In conclusion, the recognition of a peculiar phenotypic pattern of late-onset RP due to c.425A>G mutations in the *CRX* gene with a relatively mild clinical picture may be important for directing molecular testing, providing correct genetic counseling, and properly suggesting possible therapeutic options.

## Data Availability

The original contributions presented in the study are included in the article, further inquiries can be directed to the corresponding authors.
